# Bloom Helicase Along with Recombinase Rad51 Repairs the Mitochondrial Genome of the Malaria Parasite

**DOI:** 10.1128/mSphere.00718-21

**Published:** 2021-11-03

**Authors:** Payal Jha, Abhilasha Gahlawat, Sunanda Bhattacharyya, Sandeep Dey, Kota Arun Kumar, Mrinal Kanti Bhattacharyya

**Affiliations:** a Department of Biochemistry, School of Life Sciences, University of Hyderabad, Hyderabad, India; b Department of Biotechnology and Bioinformatics, School of Life Sciences, University of Hyderabad, Hyderabad, India; c Department of Animal Biology, School of Life Sciences, University of Hyderabad, Hyderabad, India; The Hebrew University of Jerusalem

**Keywords:** PfBlm, PfRad51, mitochondria, DNA repair, homologous recombination, *Plasmodium falciparum*

## Abstract

The homologous recombination (HR) pathway has been implicated as the predominant mechanism for the repair of chromosomal DNA double-strand breaks (DSBs) of the malarial parasite. Although the extrachromosomal mitochondrial genome of this parasite experiences a greater number of DSBs due to its close proximity to the electron transport chain, nothing is known about the proteins involved in the repair of the mitochondrial genome. We investigated the involvement of nucleus-encoded HR proteins in the repair of the mitochondrial genome, as this genome does not code for any DNA repair proteins. Here, we provide evidence that the nucleus-encoded “recombinosome” of the parasite is also involved in mitochondrial genome repair. First, two crucial HR proteins, namely, Plasmodium falciparum Rad51 (PfRad51) and P. falciparum Bloom helicase (PfBlm) are located in the mitochondria. They are recruited to the mitochondrial genome at the schizont stage, a stage that is prone to DSBs due to exposure to various endogenous and physiologic DNA-damaging agents. Second, the recruitment of these two proteins to the damaged mitochondrial genome coincides with the DNA repair kinetics. Moreover, both the proteins exit the mitochondrial DNA (mtDNA) once the genome is repaired. Most importantly, the specific chemical inhibitors of PfRad51 and PfBlm block the repair of UV-induced DSBs of the mitochondrial genome. Additionally, overexpression of these two proteins resulted in a kinetically faster repair. Given the essentiality of the mitochondrial genome, blocking its repair by inhibiting the HR pathway could offer a novel strategy for curbing malaria.

**IMPORTANCE** The impact of malaria on global public health and the world economy continues to surge despite decades of vaccine research and drug development efforts. An alarming rise in resistance toward all the commercially available antimalarial drugs and the lack of an effective malaria vaccine brings us to the urge to identify novel intervention strategies for curbing malaria. Here, we uncover the molecular mechanism behind the repair of the most deleterious form of DNA lesions on the parasitic mitochondrial genome. Given that the single-copy mitochondrion is an indispensable organelle of the malaria parasite, we propose that targeting the mitochondrial DNA repair pathways should be exploited as a potential malaria control strategy. The establishment of the parasitic homologous recombination machinery as the predominant repair mechanism of the mitochondrial DNA double-strand breaks underscores the importance of this pathway as a novel druggable target.

## INTRODUCTION

Malaria continues to be one of the deadliest infectious diseases of mankind in the modern era. Half of the world’s population is at risk of malaria: children and pregnant women are severely affected by this pathogen (1). Plasmodium falciparum, one out of the five species known to infect human beings, accounts for the highest mortality rate by causing cerebral malaria. The global burden of malaria infection reduced in the last decade by the use of artemisinin-based combination therapies (ACT). However, complete eradication of this life-threatening disease is still unachievable owing to the increasing cases of antimalarial drug resistance and the absence of an effective vaccine (2). Thus, the current scenario is urging us to expand our understanding of parasite biology in order to identify novel antimalarial drug targets.

In *Plasmodium*, mitochondrial biology is unconventional in several aspects. First, although the genome codes for the tricarboxylic acid cycle (TCA) enzymes, the TCA cycle is not cyclic and does not result in full oxidation of glucose to yield ATP in the blood stage of the parasite (3). Second, the protein translation machinery seems to be unusual with the highly fragmented mitochondrial rRNA genes (4–6). Third, the main purpose of the electron transport chain (ETC) appears to serve pyrimidine biosynthesis (7). Owing to these unusual features and its indispensable nature, the *Plasmodium* mitochondrion continues to be an attractive drug target. However, until now, only the *Plasmodium* ETC has been investigated extensively as a drug target (8, 9). We urge that other aspects of the mitochondrial biology of this parasite, including genome maintenance, deserve thorough exploration for the identification of novel drug targets.

Each malaria parasite contains a single mitochondrion with multiple genome copies (approximately 30 to 100 copies) (10). The mitochondrial genome of *Plasmodium* is the smallest reported thus far: it is only 6 kb long and harbors only three protein-coding genes. Several such linear units of the mitochondrial genome are arranged in tandem arrays forming concatemers of highly branched structures (11). The mitochondrial DNA (mtDNA) replication is believed to take place either via the rolling-circle mode or by involving recombination intermediates (11). Among the DNA repair mechanisms, only the base excision repair (BER) mechanism is characterized in *Plasmodium* mitochondria (12–14). In model organisms, factors involved in mismatch repair (MMR) and nucleotide excision repair (NER) have been identified in the mitochondrial extracts (15). The roles of Rad51-mediated homology-directed repair (HDR) and microhomology-mediated end joining (MMEJ or alt-EJ) have also been implicated in the repair of DNA double-strand breaks (DSBs) of human mtDNA (16–20). In this study, we have addressed whether HDR is operational in the mitochondria of P. falciparum and characterized the factors involved in it.

For the repair of the *Plasmodium* nuclear genome, both the homologous recombination (HR) pathway and the alt-EJ mechanism have been implicated (21, 22). While the factors involved in alt-EJ remain elusive, several factors of the HR pathway have been identified. The central player of HR, P. falciparum Rad51 (PfRad51), possesses the canonical ATP hydrolysis, and strand exchange activities (23). Small-molecule inhibitors that block the ATPase activity or the homomerization of PfRad51 displayed a profound effect on the DSB repair of the nuclear genome (24). In Plasmodium berghei, loss of Rad51 function resulted in complete abrogation of the DSB repair, implying that the HR pathway is the predominant DSB repair mechanism (25). Apart from Rad51, the DNA damage sensor protein P. falciparum Mre11 (PfalMre11) nuclease has also been identified and characterized (26). The important role of P. falciparum Bloom helicase, PfBlm, in the repair of the nuclear genome has recently been reported (27). This particular RecQ helicase has previously been implicated in replication, genome maintenance, and regulation of *var* gene expression in this parasite (28, 29). A chemical inhibitor of PfBlm blocked the DSB repair in this parasite and thereby rendered the parasites hypersensitive to DNA-damaging agents. Such DNA damage sensitivity can be reversed by the overexpression of PfBlm (27). PfBlm was found to interact with PfRad51, PfalMre11, and PfTopoIII (27, 30), implying the existence of a “recombinosome” complex in the parasite. PfTopoIII, a protein located in both the nucleus and mitochondria, has been found to provide a growth advantage to the hydroxyurea (HU)-arrested parasite (30).

Here, we investigated the possible role of the nucleus-encoded HR proteins in the repair of DSBs in the mitochondrial genome of this parasite. To this end, we have developed an inducible system that facilitates the creation of random DSBs in the mtDNA and allows us to study the repair process. The model additionally provided the feasibility to monitor the entry and exit of different factors before the induction of DSBs, during the repair process, and after the repair of mtDNA. Using this system, we have established that the HR proteins, particularly PfRad51 and PfBlm, are involved in the repair of mitochondrial DSBs.

## RESULTS

### Organelle localization of nucleus-encoded repair proteins PfBlm and PfRad51.

**FIG 1:**
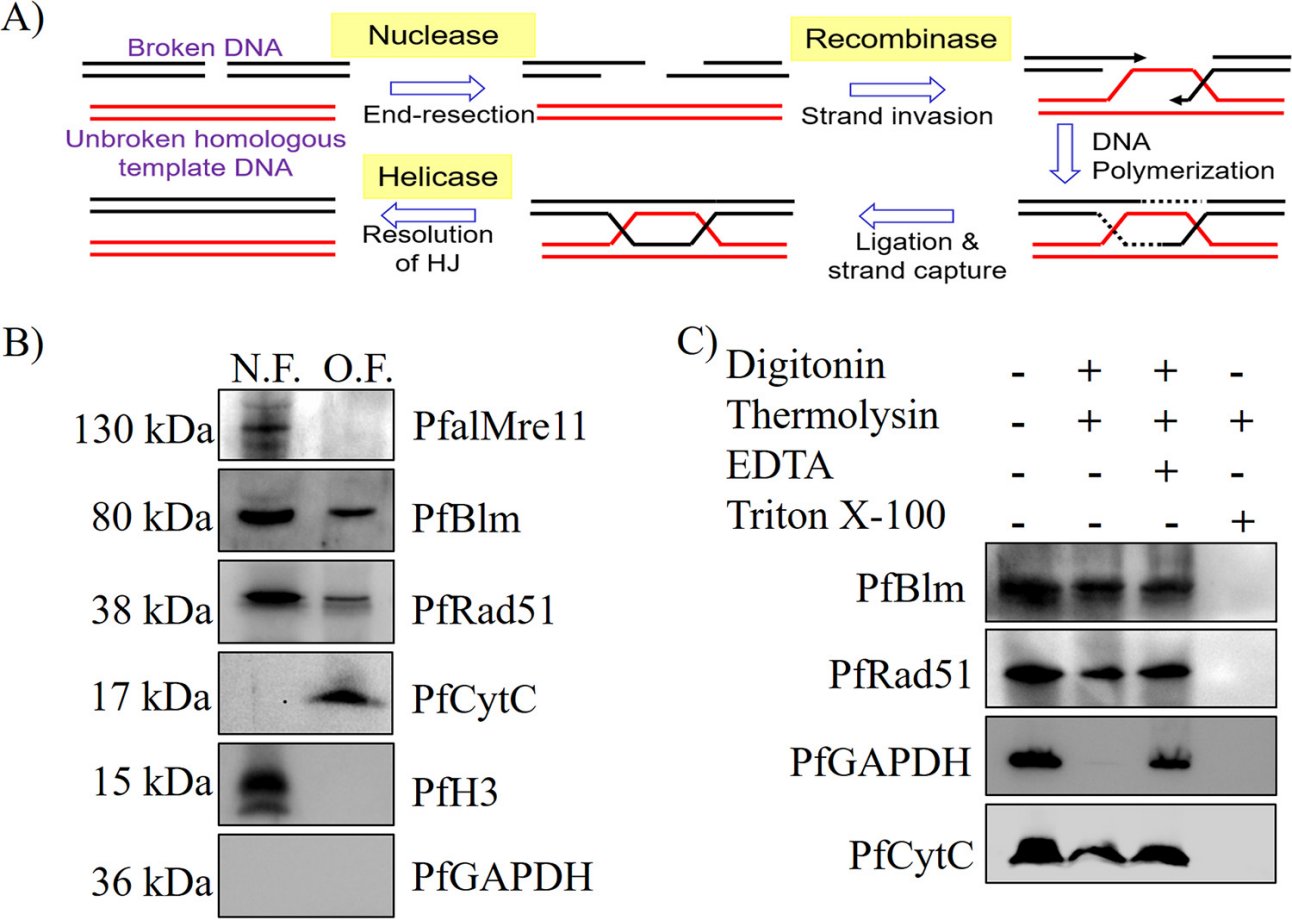
Organelle localization of nucleus-encoded repair proteins PfBlm and PfRad51. (A) Diagrammatic representation of five key steps of the homologous recombination pathway. (B) Western blot displaying the presence of PfBlm, PfRad51, and PfalMre11 in the organelle fraction (O.F.) or nuclear fraction (N.F.) using specific antibodies against these proteins. PfCytC, PfH3, and PfGAPDH serve as mitochondrial, nuclear, and cytosolic markers, respectively. The subcellular localization study was performed with parasites from the trophozoite (28 h postinfection [hpi]) stage. (C) Western blots showing the protease protection of repair proteins: PfBlm and PfRad51 were protected from thermolysin digestion upon membrane permeabilization with digitonin but not with Triton X-100. EDTA was used as a quencher of the thermolysin activity. Various treatments are marked at the top. PfGAPDH and PfCytC serve as cytosolic and mitochondrial markers, respectively.

As the mitochondrial genome of P. falciparum is vulnerable to free radicals and reactive-oxygen-species-induced DNA double-strand breaks, and this genome does not code for any DNA repair proteins, we sought to investigate whether the nucleus-encoded HR proteins are involved in the repair of such breaks. Mechanistically, HR includes five steps: DSB resection, strand invasion, DNA polymerization, branch migration followed by ligation, and resolution of the Holliday junction (HJ) (Fig. 1A). In P. falciparum, three HR proteins have been implicated to play pivotal roles in repairing DSBs in the nuclear genome: PfalMre11 (DSB resection), PfRad51 (strand invasion), and PfBlm (HJ resolution) (22, 26, 27, 30). We have investigated whether these three proteins are also localized in the mitochondria of the parasite. To this end, we have employed subcellular fractionation followed by the Western blotting technique. We performed subcellular fractionation using synchronous trophozoite stage cultures owing to the abundance of these recombination proteins in that stage (23, 26, 27). We found the presence of PfBlm and PfRad51 in both the nuclear and organelle fractions (Fig. 1B). However, PfalMre11 was exclusively located in the nuclear fraction. P. falciparum CytC (PfCytC), P. falciparum histone H3 (PfH3), and P. falciparum glyceraldehyde-3-phosphate dehydrogenase (PfGAPDH) were used as mitochondrial, nuclear, and cytoplasmic markers, respectively. These data showed organelle localization of PfBlm and PfRad51, but not PfalMre11. To ascertain their organelle localization, a thermolysin protection assay was performed. Synchronous trophozoite stage parasites were permeabilized using digitonin or Triton X-100 detergents followed by thermolysin digestion. Subsequently, the undigested proteins were assessed by Western blotting. PfGAPDH and PfCytC were used as the cytosolic and mitochondrial markers, respectively. It was found that the cytosolic marker protein was not protected from the protease digestion, whereas the mitochondrial marker protein in the digitonin-treated sample was protected from the protease digestion. A part of the aforementioned proteins in the digitonin-treated sample was found to be protected from the protease digestion, suggesting localization of these proteins in the organelle(s) lumen (Fig. 1C).

**FIG 2:**
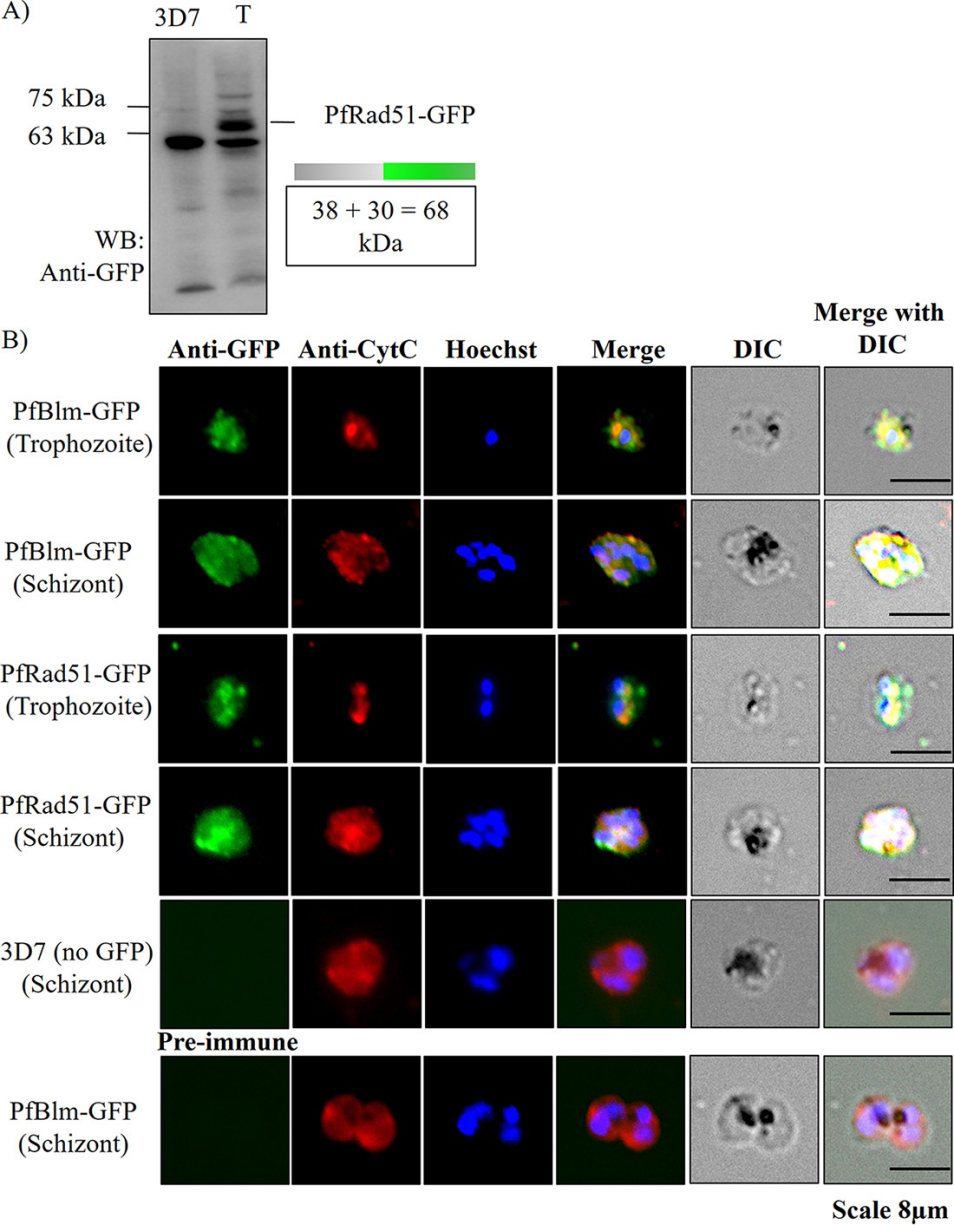
Mitochondrial localization of PfBlm and PfRad51. (A) Western blot (WB) displaying the expression of PfRad51-GFP protein in the transfected (T) parasite line and not in the untransfected (3D7) parasite line. The positions of the molecular mass markers (in kilodaltons) are indicated to the left of the blot. (B) Immunofluorescence images displaying localization of PfBlm-GFP and PfRad51-GFP (green) to mitochondria (red) and/or nucleus (blue) of Plasmodium falciparum parasite. Anti-CytC antibody and Hoechst dye were used to stain parasite mitochondria and the nucleus, respectively. IFA using anti-GFP in 3D7 and preimmune sera in PfBlm-GFP transfected parasite served as the experimental negative controls. IFA was performed with trophozoite and schizont parasites as indicated. DIC, differential interference contrast.

Furthermore, their mitochondrial localization was established using an indirect-immunofluorescence assay (IFA). To this end, we have created a transgenic parasite line expressing a C-terminally tagged PfRad51-green fluorescent protein (GFP) protein. The expression of PfRad51-GFP was first confirmed by Western blotting (Fig. 2A). We have used a previously reported parasite line for investigating the localization of PfBlm-GFP protein (27). Immunofluorescence assay was performed on formaldehyde-fixed trophozoite and schizont stage parasites using an anti-GFP antibody. Anti-CytC antibody was used as a mitochondrial marker, and Hoechst dye was used to stain the nucleus. Colocalization of signals from GFP-tagged PfRad51 or PfBlm with CytC confirmed the mitochondrion localization of PfRad51 or PfBlm in both the trophozoite and schizont stages (Fig. 2B). As expected, GFP-tagged PfRad51 or PfBlm proteins were also found in the parasite nucleus. The intracellular localization pattern of PfBlm observed in this study corroborates well with a previous report demonstrating that PfBlm is localized to both the nucleus and cytoplasm (31). The specificity of IFA was confirmed by using an untagged parasite line (3D7) and by employing preimmune sera, both of which served as a negative control and did not give any positive signal. Taken together, these results indicate the mitochondrial localization of PfRad51 and PfBlm and suggest a likely role of these proteins in the mitochondrial biology of the malaria parasite.

### Stage-specific interaction of PfRad51 and PfBlm with the mitochondrial genome.

**FIG 3:**
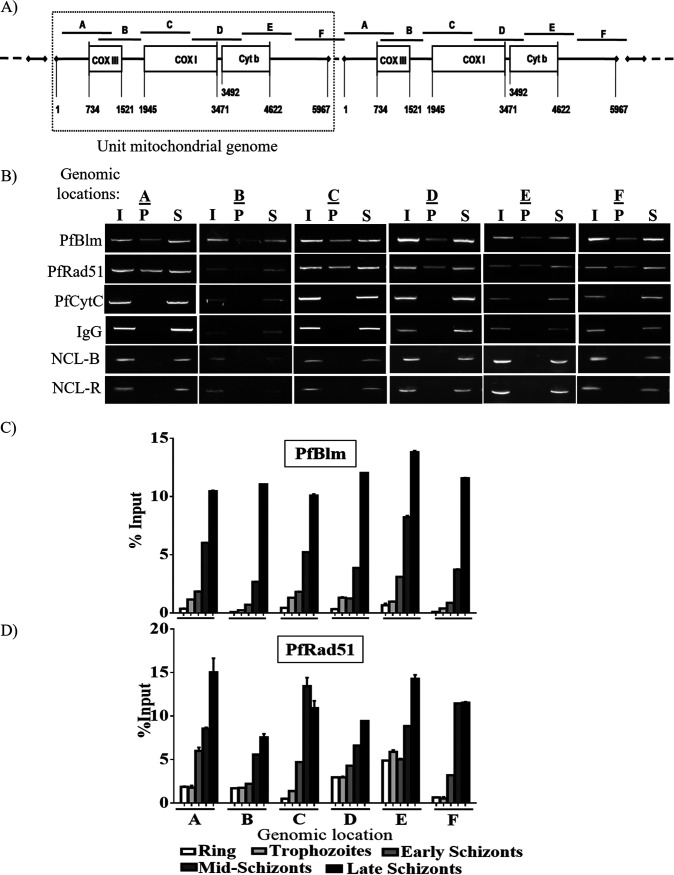
Stage-specific interaction of PfBlm and PfRad51 with the mitochondrial genome. (A) The pictographic representation of P. falciparum linear concatemeric form of 6-kb mitochondrial genome arranged in head-to-tail fashion. This mtDNA map also shows the relative positioning of three mitochondrial genes, *COXIII*, *COXI*, and *CYTB*, and six DNA fragments encompassing the entire genome, fragments A to F used in this study. (B) Agarose gels displaying the occupancy of PfBlm or PfRad51 proteins on the mitochondrial genome (fragments A to F) by mtDNA-IP, using specific antibodies against them. IP with CytC and IgG antibodies served as experimental negative controls. I, P, and S stand for input chromatin DNA, immunoprecipitated DNA, and supernatant, respectively. NCL-R and NCL-B display non-cross-linked mito-DNA IP data, using anti-PfRad51 (R) and anti-PfBlm (B) antibodies. (C and D) Relative occupancy (in terms of percent input) of PfBlm and PfRad51 as quantified by quantitative PCR (qPCR) throughout the mitochondrial genome (fragments A to F), in ring (10 to 12 hpi), trophozoite (28 hpi), early schizont (32 hpi), mild schizont (38 hpi), and late schizont (44 to 48 hpi) stages of the parasite. Values are means plus standard errors of the means (SEM) (error bars) of three repeats. Data were normalized using respective IgG-IP values.

Nuclear repair proteins are known to bind to the DNA to facilitate DNA damage repair. To investigate such interaction of PfBlm and PfRad51 with mitochondrial DNA (mtDNA), we performed mtDNA immunoprecipitation (mtDNA-IP). We have used a set of six specific primers encompassing the entire mitochondrial genome to map the association of the aforementioned proteins with mt-genome (Fig. 3A). Specific antibodies were employed to immunoprecipitate PfBlm- and PfRad51-bound mtDNA. PfCytC and IgG were used as negative controls for the mtDNA-IP experiment. To rule out any nonspecific binding of proteins upon parasite lysis, we performed mt-DNA-IP without formaldehyde cross-linking (non-cross-linked mtDNA-IP). Occupancy of PfBlm and PfRad51 was investigated with synchronous trophozoite stage parasites. Interactions of PfBlm and PfRad51 with the mtDNA were observed throughout the 6-kb genome. Further, the occupancy of PfBlm and PfRad51 at the mtDNA was investigated at different parasitic stages. We noted a gradual increase in the occupancy of both PfBlm and PfRad51 proteins at all loci with gradual maturation of the parasite’s asexual stage (Fig. 3B to D). The mature stages are not only active in DNA replication but are also expected to experience a higher propensity for DNA damage due to an increase in the metabolic activity. Thus, our finding of stage-specific occupancy of both PfRad51 and PfBlm on the mtDNA may possibly point to the involvement of these two nucleus-encoded proteins in mtDNA replication and/or repair.

### PfBlm and PfRad51 are involved in the repair of DNA double-strand breaks in the mitochondrial genome.

**FIG 4:**
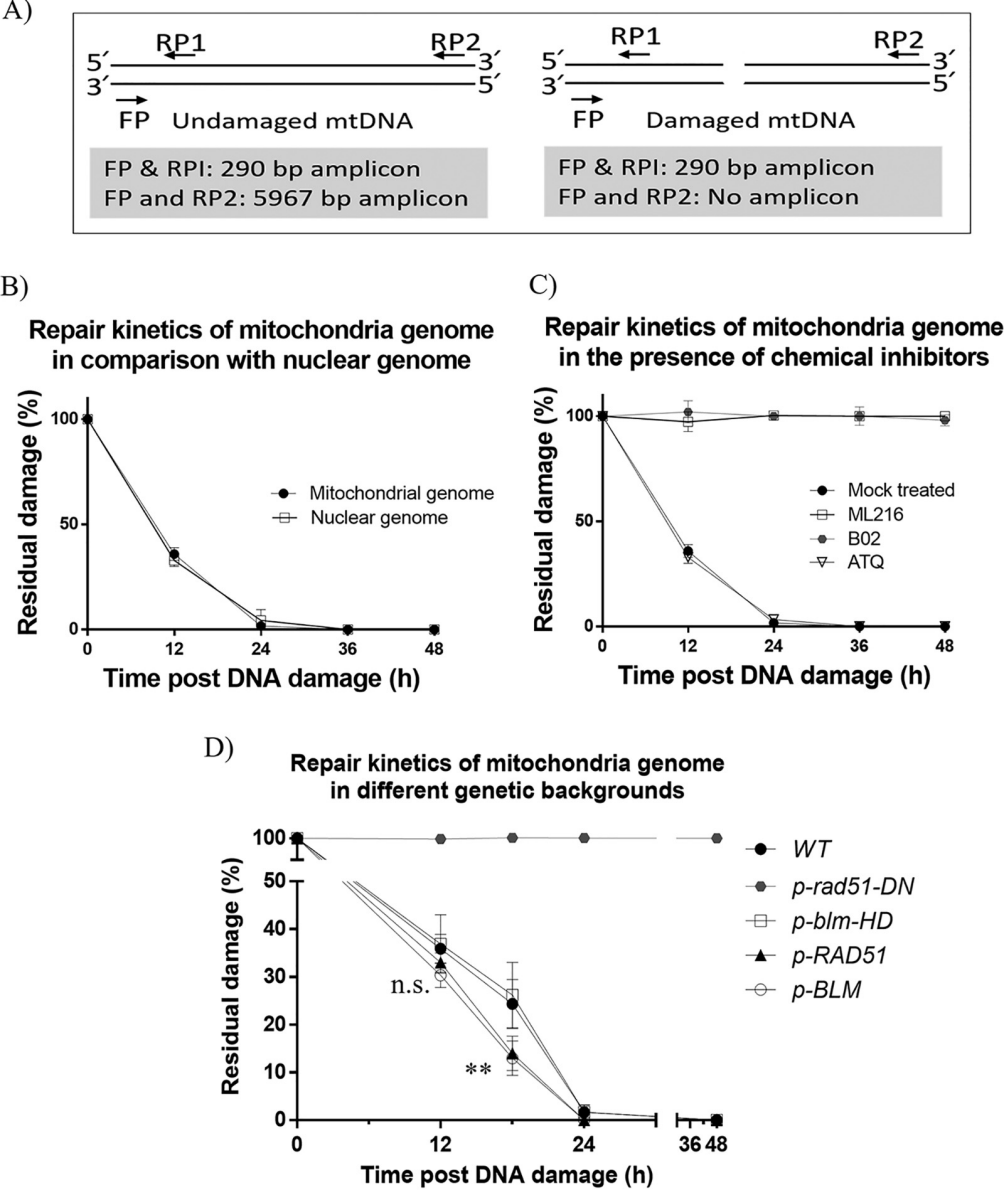
PfBlm and PfRad51 are involved in the repair of DNA double-strand breaks in the mitochondrial genome. (A) Schematic representation of the principle of mtDNA repair assay. The relative positions of the short- and long-range amplicons indicating the primer pairs are shown. (B) Parasite cultures were UV irradiated at the trophozoite stage. After DNA damage, cells were allowed to recover for 48 h. Repair of the mitochondrial DNA or nuclear DNA was assayed with specific primer sets. The percent residual damage at indicated time points was plotted using GraphPad Prism. Values are mean fluorescence intensities ± SEM (error bars) of three experimental repeats. (C) *In vitro* cultures of P. falciparum were pretreated with ML216, B02, or atovaquone (ATQ) at sublethal concentrations. The mock-treated parasite culture (grown in the drug-free complete medium) along with drug-treated parasite cultures were UV irradiated. After DNA damage, cells were grown with or without the respective drugs (ML216, B02, or ATQ) for 48 h. (D) The graph displays the kinetics of mtDNA repair in 3D7 parasite (wild type [WT]) and parasite expressing *PfRad51-GFP* (p-*PfRad51*), *PfBlm-GFP* (p-*PfBlm*), dominant negative mutant of *PfRad51K143R* (p-*rad51-DN*), or helicase-dead mutant of *PfBlmK83R* (p-*blm-HD*) from episomal plasmids. Two-tailed Student’s *t* test was used to calculate the *P* values (**, *P* value of <0.01; n.s., not significant [*P* value of >0.05]).

In order to test our hypothesis that PfRad51 and PfBlm could be involved in the repair of DSBs in the mitochondrial genome, we have employed two complementary approaches. In the first approach, we have used chemical inhibitors of these two proteins and investigated for any impairment in DNA repair activity in the mitochondrial genome. In the second approach, we have overexpressed PfRad51 or PfBlm in the wild-type parasites and investigated for any improvement in the kinetics of DSB repair in the mitochondrial genome. Blocking the repair of the nuclear genome of P. falciparum with small chemical molecules targeting PfRad51 or PfBlm has been reported earlier (24, 27). We have used these inhibitors in our study, i.e., B02 for blocking PfRad51 and ML216 for blocking PfBlm. In order to determine the working dose for UV treatment that would create measurable DSBs in the parasite genome without significantly reducing parasite viability, we have irradiated parasite cultures with different doses of UV irradiation (50, 100, 150, and 500 J/m^2^). We observed no detectable DSBs in the parasite genome when a UV dose of 50 J/m^2^ was used. Quantifiable DSBs could be observed with higher UV doses—100 J/m^2^ onwards. However, the treatment with 150- and 500-J/m^2^ UV irradiation severely compromised the parasite viability (data not shown). Therefore, in this study, we used a 100-J/m^2^ UV dose to induce DSBs. We have developed a PCR-based assay system to study the damage and its repair in P. falciparum mtDNA. A similar PCR-based method for the detection of DSBs in the P. falciparum nuclear genome has been reported earlier (27). This method is based on the principle that a longer region of the genome is likely to experience UV-induced DNA breaks and as a result, will not yield a PCR amplicon until and unless such DNA breaks are repaired. On the other hand, a very short region of the genome could be amplified even under DNA-damaging conditions as this region is less likely to harbor any break. Thus, a short-range PCR amplicon (290 bp) from the mtDNA could be used to normalize the data obtained from the long-range PCR amplicon (5,967 bp of mtDNA) (Fig. 4A). Post-UV-irradiation parasites were harvested at the 0th hour of the DNA damage and thereafter at every 12-h time interval, followed by genomic DNA isolation and PCR amplification. To this end, we have first compared the repair kinetics of the damaged mitochondrial DNA and nuclear DNA. It was observed that similar to the nuclear DNA, most of the damage of the mtDNA was repaired by 24 h after UV treatment (Fig. 4B). Next, we investigated the effect of B02 or ML216 on the kinetics of mtDNA damage repair. To this end, the parasite cultures were pretreated with the sublethal dose of the drugs (1 μM). Cells were maintained in the drug-containing medium during and after the DNA damage, and the kinetics of DNA repair was determined. Drastic abolishment of repair of mtDNA DSBs was observed in both B02-treated and ML216-treated parasite cultures (Fig. 4C). Atovaquone, a chemical inhibitor of cytochrome *bc*_1_ complex of electron transport chain, with no known effect on DSB repair, was used as a negative control. Treatment with a sublethal dose of atovaquone did not affect DNA repair, since the damaged DNA was repaired by 24 h after UV irradiation as observed for the mock-treated parasites (Fig. 4C). The long-range PCR product of this assay was sequenced to ascertain that the amplicons were mitochondrial genome specific (data not shown).

Our hypothesis predicts that overexpression of these two DSB repair proteins would have a positive effect on DSB repair. In order to test that, we have created two parasite lines each expressing either the *PfRAD51* or *PfBLM* gene from an episomal plasmid. We have compared the repair kinetics of the wild-type 3D7 strains and the two parasite lines harboring extra copies of either of the genes (p-*RAD51* and p-*BLM*). Significantly faster repair kinetics were observed for each of the two transgenic parasite lines. It was observed that at the end of 18 h after DNA damage, in the wild-type 3D7 strain, only 74% repair was achieved, but in the transgenic strains, 86 to 87% repair was completed (Fig. 4D). The experiment was repeated more than three times with transgenic parasite strains, and we found that the increase in repair efficiency in both *PfBLM-* and *PfRAD51*-expressing strains are statistically significant. In order to ascertain that such increased efficiency is due to expression of the additional copies of the respective genes, we have created two more parasite lines expressing the mutant versions of the genes. Earlier it was reported that the helicase-dead mutant of *PfBLM* (*Pfblm-K83R*) could not confer any survival advantage to the methyl methane sulfonate (MMS)-treated parasites as opposed to the parasites expressing additional copies of the wild-type *PfBLM* gene (27). We have included this parasite line in our study as a control. Similarly, the expression of a mutant version of *PfRAD51* (*PfRad51K143R*) had a dominant negative effect on the wild-type *PbRAD51* and rendered such parasites more vulnerable to MMS treatment (25). For this study, we have created the *PfRAD51* dominant negative P. falciparum line by expressing the *PfRad51K143R* mutant allele from an episomal plasmid PfCENv3. This parasite line has also served as a control for our experiments. It was observed that the *Pfblm-HD*-expressing parasite line did not confer any faster repair of mtDNA. As expected in the *Pfrad51-DN* line, the DSB repair was completely abolished (Fig. 4D). Taken together, our findings establish the pivotal roles of the nucleus-encoded HR proteins in the DSB repair of the mitochondrial genome.

### DNA damage induces organelle translocation and mtDNA recruitment of PfRad51 and PfBlm.

**FIG 5:**
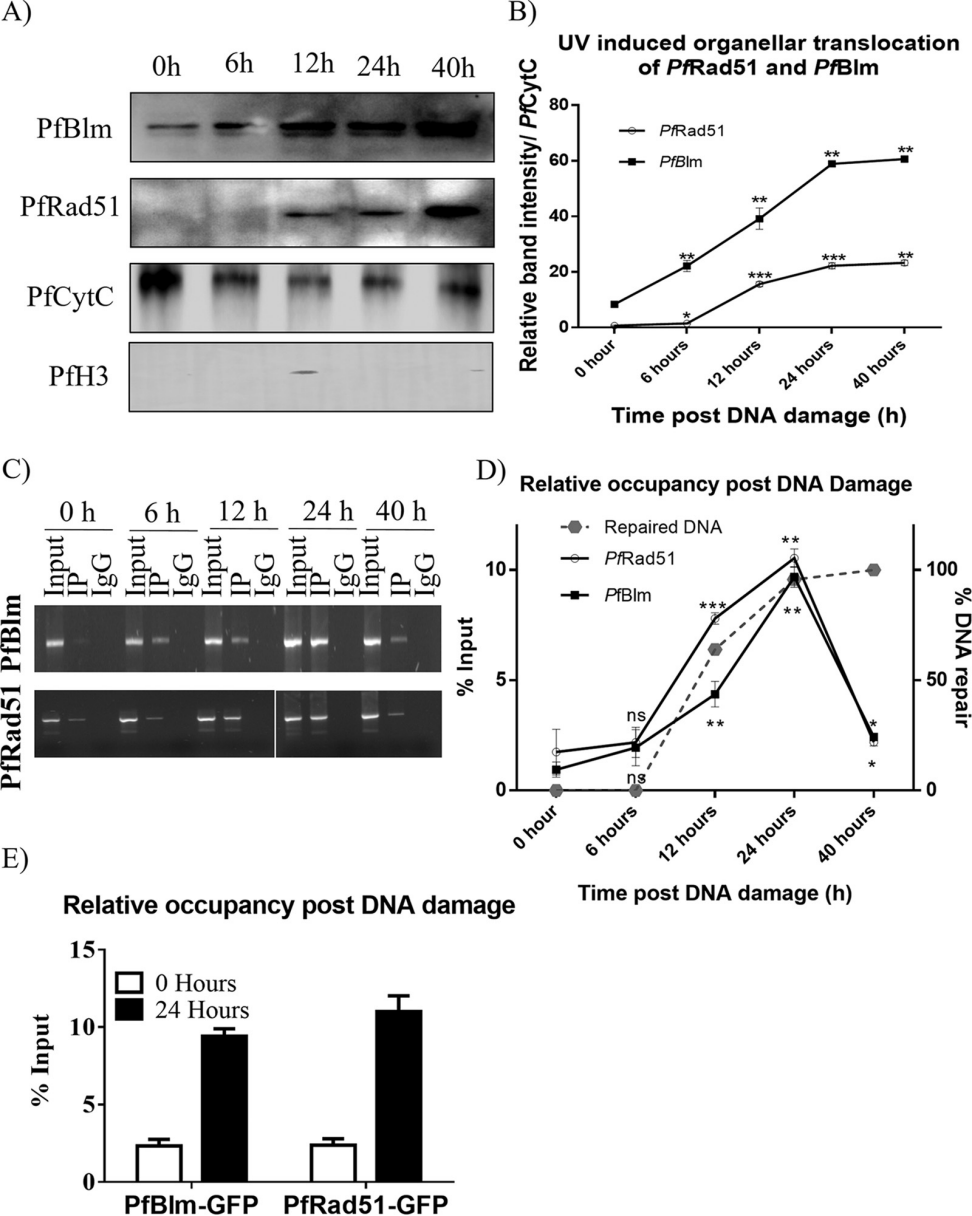
DNA damage induces organelle translocation and mtDNA recruitment of PfRad51 and PfBlm. (A) A representative Western blot showing the levels of PfBlm and PfRad51 proteins in the organelle fraction at respective time points after DNA damage. Tightly synchronous trophozoite stage parasites were used for this assay. The mitochondrial marker PfCytC was used as a loading control. The nuclear marker PfHistone 3 (PfH3) shows the purity of the organelle fraction. (B) Band intensities of PfBlm and PfRad51 were normalized using PfCytC. Image J software was used to quantify the band intensities in the Western blots of the three experimental repeats. Data are mean band densities ± SEM (error bars). Two-tailed Student’s *t* test was used to calculate the *P* values (*, *P* value of <0.05; **, *P* value of <0.01; ***, *P* value of < 0.001). (C) Agarose gels represent PCR amplification of the mitochondrial locus A in the following samples: input chromatin (Input), PfBLM- or PfRad51-immunoprecipitated chromatin (IP), and rabbit immunoglobulin G immunoprecipitated chromatin (IgG) at the indicated time points after UV irradiation. The upper and lower panel displays the recruitment of PfBlm and PfRad51 proteins to mtDNA post DNA damage. (D) The plot displays the kinetics of the relative occupancy of PfBlm and PfRad51 to mtDNA (locus A) upon DNA damage. The precipitated mtDNA was quantified by qPCR and is expressed in terms of percent input in the left-hand *y* axis. Data were normalized against respective IgG-IP values. The repair kinetics of the damaged mtDNA is represented as the dashed line on the right-hand *y* axis. (E) The bar graph represents the DNA damage-induced enrichment of GFP-tagged PfBlm and PfRad51 to mtDNA at 0 and 24 h post-UV treatment. Values are means ± SD (error bars) (*n* = 3). *, *P* value of <0.05; **, *P* value of <0.01; ***, *P* value of <0.001; n.s., not significant (*P* value of >0.05).

We investigated the organelle import of PfRad51 and PfBlm in response to the UV-mediated DSBs. We reasoned that if these two proteins are directly involved in repairing the mitochondrial DSBs, they are likely to be imported into the mitochondria and then recruited at the damaged mtDNA in response to UV irradiation. To this end, P. falciparum cultures at the trophozoite stage were subjected to 100-J/m^2^ UV radiation to induce DSBs. Parasites were collected at 0 h, 6 h, 12 h, 24 h, and 40-h time points after UV irradiation, and the organelle fractions were isolated. Proteins from the organelle fractions were subjected to Western blot analysis using specific antibodies against PfRad51 or PfBlm protein. A gradual increase in both proteins was observed up to the 12th h, and then a sharp increase was observed at the 24-h time point, which remained almost unaltered thereafter (Fig. 5A and B). The mitochondrial protein PfCytC, which is also nucleus encoded and imported to the mitochondria, acted as a loading control as it is not related to the DNA damage response. The nuclear protein PfH3 acted as a negative control for this experiment. Thus, our findings establish that DNA damage induces increased organelle localization of repair proteins, Pablum and PfRad51.

Further, we investigated whether the increased import of PfRad51 and PfBlm is correlated with increased occupancy of these proteins on damaged mtDNA. To this end, we have determined the time kinetics of the recruitment of PfRad51 and PfBlm at the mtDNA by performing mtDNA-IP experiments. The relative occupancy of PfBlm and PfRad51 proteins on mtDNA were found to be increased significantly after 12 h post-DNA damage, with no significant change during the initial 6 h. The recruitment of these HR proteins was further seen to be significantly elevated, and at the end of 24 h, it reached a peak. Thereafter, the occupancy of these repair proteins was reduced to the basal level at the end of the 40-h time point (Fig. 5C and D). Rivetingly, the pattern of the recruitment kinetics was found to be parallel with the DNA repair kinetics, and the peaks of both the kinetics coincided at the 24th h post-DNA damage (Fig. 5D). These findings provide evidence for the direct role of PfRad51 and PfBlm in the repair of damaged mtDNA. To validate the faster repair kinetics exhibited by the parasite lines ectopically expressing GFP-tagged PfRad51 or PfBlm, we investigated the recruitment of both recombinant proteins to mtDNA at 0 and 24 h post-UV irradiation (Fig. 5E). As expected, we noted a markedly enhanced recruitment of both the proteins at 24 h post-DNA damage. Taken together, our findings suggest that in response to mtDNA damage, PfRad51 and PfBlm translocate to the mitochondria and are recruited at the damaged sites in mtDNA during the first 12 to 24 h, a duration that coincides with the time required for the completion of the mtDNA repair. However, once the damage is repaired, these proteins are dislodged from the DNA but still reside within the mitochondria, at least up to the 40-h time point.

### Mitochondrial localization and mtDNA interaction of putative mitochondrial DNA polymerase.

**FIG 6:**
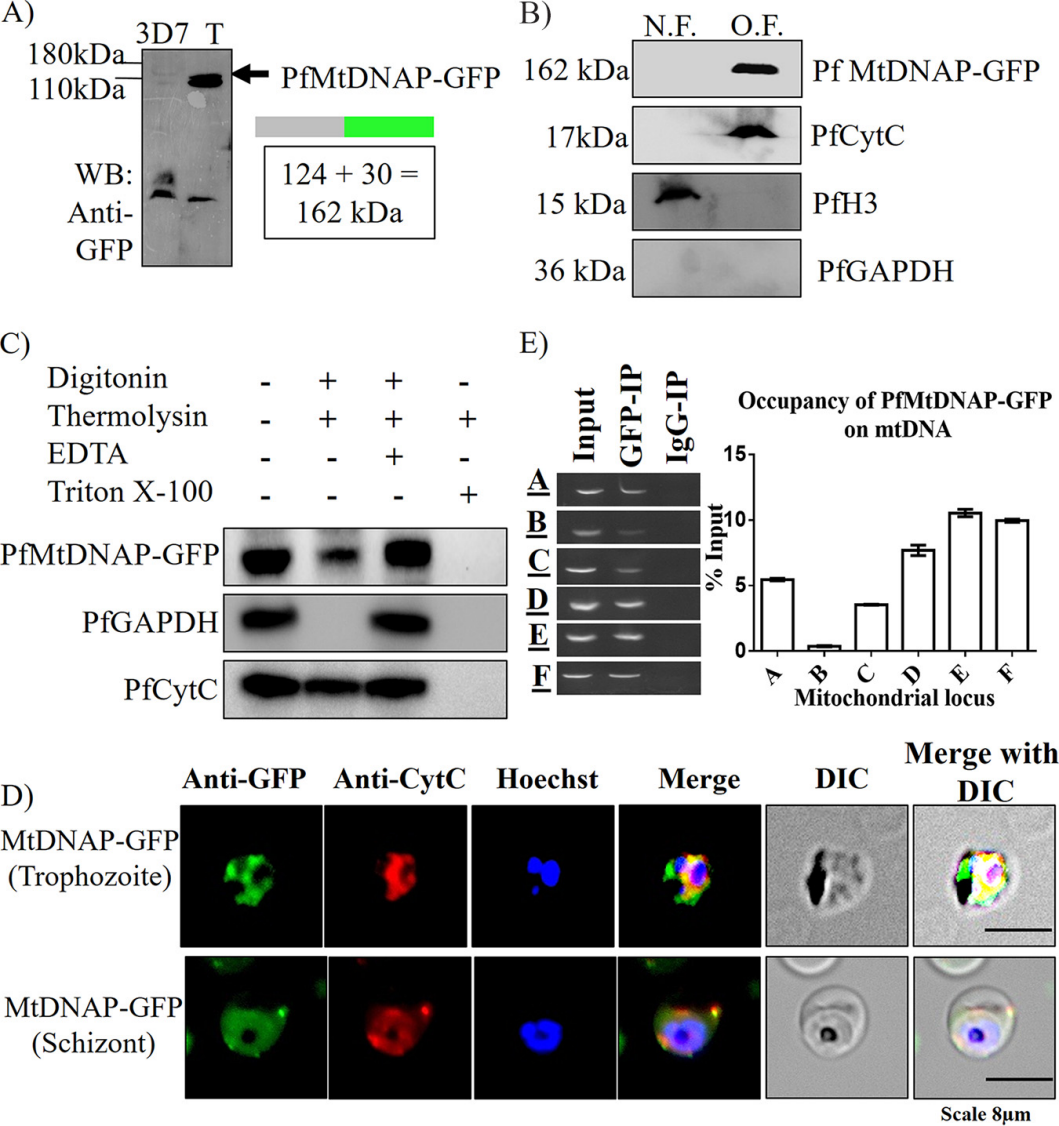
Mitochondrial localization and mtDNA association of putative PfMtDNAP. (A) Western blot demonstrating episomal expression of C-terminal GFP-tagged PfMtDNAP in transfected parasite (3D7, control parasite; T, transfected parasite). (B) Demonstration of PfMtDNAP-GFP in the organelle fraction (O.F.) but not in the nuclear fraction (N.F.) of P. falciparum using anti-GFP antibody. PfCytC, PfH3, and PfGAPDH serve as mitochondrial, nuclear, and cytosolic markers, respectively. (C) Western blots represent the thermolysin protection of PfMtDNAP-GFP upon differential permeabilization with digitonin or Triton X-100. PfGAPDH and PfCytC served as cytosolic and nuclear markers, respectively. (D) Immunofluorescence assay showing colocalization of PfMtDNAP-GFP signal (green) with mitochondrial marker PfCytC signal (green). IFA was performed with trophozoite and schizont stage parasites. (E) Agarose gels depict the association of PfMtDNAP-GFP protein with the mitochondrial genome (A to F loci) by mtDNA-IP, using anti-GFP antibody. IP with IgG antibody served as experimental negative controls. Here, Input, GFP-IP, and IgG-IP denote Input chromatin DNA, anti-GFP immunoprecipitated DNA, and anti-IgG immunoprecipitated DNA, respectively. The bar graph displays the relative occupancy of PfMtDNAP-GFP on mtDNA fragments A to F in terms of percent input. Data were normalized using respective IgG-IP values.

As mentioned earlier that during the third step of HR (Fig. 1A), the invaded strand is extended by a coordinated action of DNA polymerase, recombinase, and helicase, we sought to investigate whether PfRad51 and PfBlm are also engaged with the mitochondrial DNA polymerase (mtDNAP). The *Plasmodium* gene PF3D7_0625300 harboring a type A polymerase domain is predicted to be a mitochondrial DNA polymerase in P. falciparum owing to a very high score by the software predicting mitochondrial localization (Mitoport-II score = 96%). This gene was annotated as a putative mtDNAP due to the lack of biochemical and genetic studies. In a previous study, this gene was identified as a member of the type A family DNA-dependent DNA polymerase based on conserved domain analysis (32). The conserved catalytic domain of *PfDNAP*, human DNA polymerase gamma, and Saccharomyces cerevisiae DNA polymerase gamma fall under type A DNA polymerase. In model eukaryotes, DNA polymerase gamma (Polγ/PolG) holds sole responsibility for both mtDNA replication and repair (33, 34). DNA PolG-like activity has been biochemically characterized in P. falciparum (35); however, the gene coding for such activity remained elusive. We sought to investigate the cellular localization of the putative P. falciparum mtDNAP (PfMtDNAP). To this end, we created a parasite line harboring GFP-tagged PfMtDNAP. Protein expression was checked in the transfected parasite line (Fig. 6A). We investigated its organelle(s) localization exploiting cellular fractionation followed by Western blotting (Fig. 6B). Organelle localization was further validated by protease protection assay. Indeed, the putative PfMtDNAP was found to be exclusively localized in the organelle fraction (Fig. 6C). Additionally, the results of the immunofluorescence assay also confirmed the mitochondrial localization of this protein (Fig. 6D). Being a DNA-dependent DNA polymerase, PfMtDNAP must associate itself with the mitochondrial genome. Therefore, we inspected the recruitment of this protein on mtDNA using anti-GFP mtChIP. In agreement with our hypothesis, PfMtDNAP was found to be associated with the mtDNA (Fig. 6E). Unlike PfRad51 and PfBlm, the mtDNA interaction of PfMtDNAP was found to be more predominant with the second half of the mt-genome (loci E and F). Taken together, it appears that PF3D7_0625300 encodes a DNA polymerase that is likely targeted to the parasite’s mitochondria.

### Homologous recombination proteins interact with mitochondrion-specific DNA polymerase.

**FIG 7:**
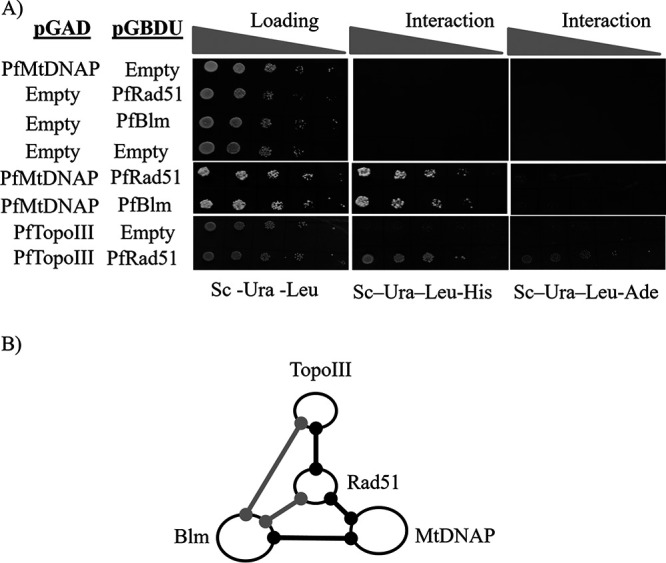
Homologous recombination proteins interact with mitochondrion-specific DNA polymerase. (A) Yeast two-hybrid analysis showing interactions between different parasite proteins. PfBlm and PfRad51 were fused with the GAL4 DNA binding domain of the bait vector pGBDU-C1. Likewise, PfMtDNAP and PfTopoIII were fused with the GAL4 activation domain (GAL4-AD) of the prey vector pGAD-C1. Interactions of the fusion proteins were examined in PJ69-4A yeast strain containing *ADE2* and *HIS3* reporter genes under the *GAL4* promoter. An equal number of transformed yeast cells and their 10-fold serial dilutions were spotted on synthetic complete medium lacking uracil and leucine (Sc -Ura -Leu) agar plates to establish the expression of fusion proteins. Simultaneously, yeast cells were spotted on synthetic complete medium lacking uracil, leucine, and histidine (Sc–Ura–Leu-His) and synthetic complete medium lacking uracil, leucine, and adenine (Sc –Ura–Leuc -Ade) triple dropout agar plates to assess the protein-protein interactions. Interaction of empty prey vector and PfBlm or PfRad51. Empty bait vector and PfMtDNAP or PfTopoIII were used as experimental negative controls. (B) A pictorial representation of the putative “recombinosome” complex involved in the repair of mtDNA. The black connecting lines represent protein-protein interactions established in this study, whereas the gray connecting lines represent previously reported protein interactions.

We investigated whether PfRad51 and PfBlm interact with the putative PfMtDNAP as it is reasonable to propose that the recombinase and helicase must engage themselves with other proteins in order to carry out homology-directed repair of the damaged mtDNA. A yeast two-hybrid assay was employed for protein-protein interaction studies. *PfBLM* or *PfRAD51* was cloned in the bait vector containing the *GAL4* DNA binding domain and *URA3* selectable marker. PfMtDNAP was cloned in the prey vector harboring *GAL4* activation domain and *LEU2* selectable marker. The cotransformed PJ694A yeast cells were analyzed on synthetic complete agar plates (lacking leucine, uracil, and histidine or lacking leucine, uracil, and adenine) for the expression of reporter gene *HIS3* or *ADE2*, where expression of *HIS3* scores for both strong and weak interactions and *ADE2* scores for only strong interactions. It was observed that the interaction between repair proteins and PfMtDNAP was weak in nature (Fig. 7A). We further investigated the interaction between PfRad51 and PfTopoIII, as PfTopoIII was reported to be localized in P. falciparum mitochondria (28). A strong interaction was observed between PfRad51 and PfTopoIII (Fig. 7A). Earlier, both PfTopoIII and PfRad51 were found to interact with PfBlm (27, 30). Thus, it is likely that PfMtDNAP, PfRad51, PfBlm, and PfTopoIII could be parts of a multiprotein complex (Fig. 7B). Our localization studies reiterate the possibility that such a complex may exist within the mitochondria.

## DISCUSSION

Here, we established that P. falciparum possesses a functional DSB repair mechanism for its mitochondrial genome. First, two prominent nucleus-encoded HR proteins, PfRad51 and PfBlm, are present in the mitochondria, in addition to their nuclear localization. These proteins are associated with the mtDNA, particularly at the DNA damage-prone matured stages of the asexual parasites. Second, the chemical inhibitors of PfRad51 or PfBlm abrogated the repair of UV-induced DSBs of the mtDNA. A genetic study with a dominant negative mutant of PfRad51 also corroborated this finding. Third, the recruitment of these two proteins followed the same kinetics of the mtDNA repair. Both PfRad51 and PfBlm were found to exit the mtDNA once the repair is done. Finally, PfRad51 and PfBlm were found to be associated with the mitochondrial DNA polymerase PfMtDNAP, and topoisomerase PfTopoIII, which was previously reported to be associated with the mtDNA (28). PfTopoIII provides a survival advantage to the HU-treated parasites where HU is responsible for causing DNA damage and replication blockage. Thus, this is the first demonstration that suggests *Plasmodium* tDNA is not refractory to DSB repair.

Compared to the nuclear genome, the mtDNA is more prone to oxidative damages, primarily due to its proximity to the ETC. Additionally, the absence of the protective histone proteins makes the mtDNA even more vulnerable to DNA-damaging agents. In a living human being, the estimated rate of point mutations in the mitochondrial genome is 6 × 10^−8^ per base pair per year which is 100-fold higher than the nuclear somatic point mutation rate (36). A common belief that persisted in the scientific field for decades, without any substantial data, was that the damage on the mtDNA remains unrepaired. Such a belief originated from the observation that there was no removal of UV-induced pyrimidine dimers from the mtDNA (37). It was later found that the mtDNA containing the damaged bases was confined to a fragmented subset of the total complement (38). With the discovery of several factors belonging to the BER pathway in the mitochondria of most model organisms (39, 40), it is now well-established that BER is a predominant repair mechanism in the mitochondria. In addition to the BER, the HR mechanism mediated by Rad51 and its paralogues is also found to be operational in yeast and humans (16–18). There is a proposition that microhomology-mediated end joining (alt-EJ) could also be an important DSB repair mechanism of the mtDNA in model organisms (19, 20). In P. falciparum, two apurinic/apyrimidinic (AP) endonucleases have been found in the mitochondria (12–14), suggesting the existence of the BER mechanism in the parasite mitochondria. Even though the alt-EJ mechanism is discovered in this parasite (21), it is currently unknown whether this DSB repair mechanism contributes to the repair of mtDNA. Our finding that in the absence of a functional HR mechanism, there was a complete abrogation of mtDNA repair not only establishes that the HR pathway is operational in the mitochondria of malaria parasites but also suggests that it is probably the main DSB repair mechanism and that its functional absence cannot be compensated for by other DSB repair mechanism.

Rad51 and its paralogue XRCC3 have been implicated in the replication of human mtDNA (18). The schizont stage-specific association of PfRad51 and PfBlm with the mtDNA might indicate a plausible role of these two proteins in mtDNA replication, owing to the fact that the mtDNA replication coincides with this stage. However, such an association could simply arise due to an increase in replication error-induced DSBs at this stage. This unresolved question could be addressed in the future, especially because it has long been proposed that the unusual nature of *Plasmodium* tDNA replication is likely to involve recombination intermediates (11).

Despite the presence of effective BER, and HR mechanisms, P. falciparum mtDNA is prone to mutational alterations. As HR represents a faithful DNA repair mechanism that ensures genomic integrity, it is currently unclear how the mtDNA tends to accumulate more mutations than the nuclear genome. Whether or not an efficient mismatch repair (MMR) pathway is operational in the *Plasmodium* mitochondria is currently not known. Our finding that both PfRad51 and PfBlm engage themselves with the mitochondrial DNA polymerase, PfMtDNAP, raises another possibility. It could be possible that the proofreading activity of PfMtDNAP is compromised, leading to an increased rate of misincorporation during DNA replication and repair. An in-depth biochemical characterization of PfMtDNAP would be able to test this possibility.

In mammalian cells, the RecQ4 helicase, but not the Blm helicase, has been implicated in the maintenance of mtDNA (41, 42). Additionally, it was found that the mitochondrial helicase Twinkle plays a key role in the loading of Rad51 and its paralogue XRCC3 onto the mtDNA (18). The PfTwinkle helicase has been identified, and it was observed that it is located exclusively in the apicoplast organelle, but not in the parasite mitochondria (43). We found that in P. falciparum, the RecQ helicase PfBlm is involved in the maintenance of the mtDNA. Whether additional helicases are involved in this process remains elusive at this point.

Except for PfMtDNAP, all other proteins investigated in this study lack any kind of mitochondrial localization signals. Yet, PfRad51, PfBlm, and PfTopoIII, all are imported into the mitochondria. This reiterates our inadequate knowledge of parasite mitochondria import pathways. Currently, it is not clear whether the mitochondrial membrane harbors multiple import machinery that aid in translocation of proteins bearing or lacking canonical mitochondrial localization signals. Our observations that DNA-damaging agents prompt inducible translocation of different molecular-sized proteins (PfRad51 [38 kDa] and PfBlm [80 kDa]) into mitochondria can serve as a model for future investigations about mitochondria import mechanisms in this parasite.

Although *Plasmodium* mitochondrion is not the powerhouse of the cell, it is still an essential organelle. Its ETC is an indispensable process, primarily for the pyrimidine biosynthesis pathway, and probably for other metabolic pathways (7). Thus, the ETC as a whole and more particularly the mtDNA-encoded PfCytB emerged as a lucrative drug target. While increased resistance to atovaquone somehow dampened the prospect of PfCytB as a drug target, the low frequency of resistance to ELQ-300 is encouraging (44). Thus, there is renewed interest in targeting *Plasmodium* mitochondria. It is not unreasonable to propose that targeting the DSB repair mechanism of the *Plasmodium* mitochondria is likely to have deleterious consequences for the maintenance of the mitochondrial genome of this parasite. This may eventually impact mitochondrial biology and the survival of the parasites. As only the maternal mitochondrion is present in the zygote that cannot be complemented by the paternal copy, we speculate that any aberration of the mtDNA due to the inhibition of the DSB repair pathway is likely to have a profound effect on the parasites during the mosquito stages, including the transmission biology. Such a hypothesis needs to be tested in the future.

## MATERIALS AND METHODS

### Inhibitors.

Atovaquone, B02, and ML216 were purchased from Sigma-Aldrich. ML216 and B02 were dissolved in dimethyl sulfoxide (DMSO). Atovaquone was dissolved in Milli-Q water.

### P. falciparum *in vitro* culture.

P. falciparum wild-type strain (3D7) or transfectant parasite strains were cultured by the conventional candle jar method. Culture were maintained at 5% hematocrit in human erythrocytes in complete medium RPMI 1640 (Himedia) containing 1% (wt/vol) Albumax and 0.005% (wt/vol) hypoxanthine.

### Plasmid constructs.

Genomic DNA from P. falciparum 3D7 parasite strain was used as the template for gene amplification. For yeast two-hybrid assay, the full length of *PfBLM* and *PfRAD51* were cloned into the bait vector pGBDU between BamHI and SalI restriction sites. Similarly, full-length *PfMtDNAP* and *PfTOPOIII* were cloned into the prey vector pGADC1. The bait and prey vectors are 2μm yeast plasmids containing *URA3* and *LEU2* selectable markers, respectively. This resulted in the expression of fusion proteins where the N-terminal regions of *Pf*Blm and *Pf*Rad51 were fused with the *GAL4* DNA binding domain and *Pf*MtDNAP and *Pf*TopoIII were fused to the *GAL4* activation domain.

For immunofluorescence assay, *PfRAD51*, *PfBLM*, and *PfMtDNAP* were tagged with C-terminal GFP by cloning full-length genes into the pARL-GFP vector (27). For overexpressing the dominant negative mutant allele *PfRad51* (*K143R*) or the wild-type *PfRad51*, genes were cloned into episomal overexpression plasmid pPfCENv3 having blasticidin (BSD) drug resistance marker.

### Subcellular fractionation of P. falciparum culture.

Synchronous P. falciparum 3D7 cultures were treated with 0.15% (wt/vol) saponin to obtain red blood cell (RBC)-free parasite. The standard protocol for subcellular fractionation was used with slight modifications as described earlier (45). Briefly, the parasite pellet was washed with buffer I (containing 0.34 M sucrose, 15 mM NaCl, 0.2 mM EDTA, 15 mM Tris-HCl [pH 7.4], 0.2 mM phenylmethylsulfonyl fluoride). The parasite was then resuspended in buffer II (buffer I containing 0.1% Triton X-100) and homogenized using a Dounce homogenizer. The nuclear pellet was first separated by centrifuging the homogenate at 600 × *g* for 10 min. Supernatant was centrifuged at 12,000 × *g* for 30 min to obtain organelle pellet. The nuclear and organelle pellets were washed thrice with buffer I.

### Thermolysin protection assay.

For thermolysin protection assay, a previously described methodology was used with minor modifications (46). Briefly, synchronous trophozoite parasites were lysed using saponin and washed with phosphate-buffered saline (PBS). The parasite cultures were then divided into four parts and resuspended into assay buffer (50 mM HEPES-NaOH [pH 7.4], 5 mM CaCl_2,_ 300 mM sorbitol) containing no-detergent control, 0.05% digitonin, 0.05% digitonin with 10 mM EDTA or 1% Triton X-100. After incubation on ice for 10 min, thermolysin was added (25 μg per 1 mg parasite protein) and incubated on ice for 1 h. Reactions were stopped by adding 4× Laemmli buffer, samples were boiled and separated by sodium dodecyl sulfate-polyacrylamide gel electrophoresis (SDS-PAGE) followed by Western blotting.

### Transfection in P. falciparum.

Tightly synchronous ring stage-specific P. falciparum 3D7 culture with 8 to 10% parasitemia were transfected with different plasmid constructs as described previously (27). Briefly, DNA was resuspended in cytomix solution (10 mM K_2_HPO_4_ [pH 7.6], 120 mM KCl, 0.15 mM CaCl_2_, 25 mM HEPES [pH 7.6], 2 mM EGTA [pH 7.6], 5 mM MgCl_2_) and incubated at 4°C overnight. Bio-Rad Gene Pulser was used to electroporate 100 μg DNA. Transfected cells were first maintained in complete medium until the parasite reaches a parasitemia of 4% and then were maintained in the respective drug-containig medium.

### Yeast two-hybrid analyses.

The previously described procedure was used for yeast two-hybrid analyses (25). To score the interaction between the fusion proteins with activation domain and DNA binding domain, the plasmid constructs were transformed in PJ69-4a yeast cells and then assessed by growing 10-fold serial dilutions of the yeast cultures on synthetic complete agar lacking leucine, uracil, and histidine (SC−Ura−Leu−His) or lacking leucine, uracil, and adenine (SC−Ura−Leu−Ade) drop out plates for 3 to 5 days at 30°C.

### PCR-based method to quantify mitochondrial DNA damage.

The ring stage parasites were pretreated with a sublethal dose of the inhibitors, such as ML216 (1 μM), B02 (1 μM), and atovaquone (0.3 nM). Pretreated parasites were irradiated with 100-J/m^2^ UV light at the trophozoite stage. After DNA damage, the mock-treated and drug-treated parasites were cultured in the respective drug-containing medium for 48 h. Samples were collected before and after UV treatment at every 12-h interval. Genomic DNA was isolated from harvested parasites, and the amount of total DNA was quantified using the SYBR green I dye-based standard plot method. An equal amount of DNA was used as a template to amplify long- and short-range fragments. Primer set OMKB540 (5՛-GAG TGG ATT AAA TGC CCA GCC-3՛) along with OMKB541 (5՛-ATT GTT CTA CAT TAC GAG ATA CC-3՛) and OSB251 (5՛-GAC GCA TCT CTA CAA ACT ACA GAG-3՛) with OSB252 (5՛-GAC TTA TTC TGA ATA GAA TAA GAA CTC-3՛) were used to amplify mitochondrion-specific long (5,967-bp) and short (290-bp) PCR products, respectively. The long PCR products were subjected to DNA sequencing to ascertain that they are derived from the mt-genome. Further, the amount of DNA in the PCR products was quantified using SYBR green I dye. Fluorescence intensities from the short-range PCR products were used to normalize the data. The equation used to calculate percent DNA damage at any given time was 1 − (fluorescence intensity of long-range PCR product/fluorescence intensity of short-range PCR product) × 20.57 × 100, where 20.57 represents the ratio of long-range PCR amplicon size and short-range PCR amplicon size. DNA damage was considered 0% in UV-untreated control and 100% in 0 h post-UV sample. The GraphPad Prism software was used to plot the percent residual damage at each time point. The mean fluorescence intensity ± standard error of the mean (SEM) are shown (*n* = 3).

### Chromatin immunoprecipitation assay.

ChIP experiments were performed by the method of Tabassum et al. (47). Briefly, synchronous asexual stages (rings, trophozoites, and early, mid, and late schizonts) were harvested and treated with saponin to break open the RBCs. The parasite pellet was then cross-linked using 37% formaldehyde (final concentration of 0.5%). Cross-linked parasites were lysed by homogenization using Dounce homogenizer (200 strokes for rings and 100 strokes for trophozoites or schizonts). In parallel experiments, parasites without the formaldehyde cross-linking were processed similarly and acted as a negative control. The DNAs were sheared using an Elma water bath sonicator to generate the chromatin fragments of an average size of 1 kb. The fragmented DNAs were run on an agarose gel to verify the size distributions. Antibodies against *Pf*Rad51 and *Pf*Blm were used to specifically immunoprecipitate the DNA-protein complex. Anti-CytC and anti-IgG (rabbit immunoglobin G antibody) were used as negative controls for the ChIP experiments. The precipitate was treated with 5 M NaCl to reverse cross-link the proteins, and finally, the DNA was extracted using the phenol-chloroform method. Recruitment of proteins throughout the mitochondrial genome was assessed using six pairs of primers: OMKB540 (5՛-GAG TGG ATT AAA TGC CCA GCC-3՛) and OSB251 (5՛-GAC GCA TCT CTA CAA ACT ACA GAG-3՛) for fragment A, OMKB614 (5՛-CAT AAC ATT TTT TAG TCC CAT GC-3՛) and OSB252 (5՛-GAC TTA TTC TGA ATA GAA TAA GAA CTC-3՛) for fragment B, OMKB615 (5՛-CTG GCC TAC ACT ATA AGA ACG-3՛) and OSB176 (5՛-CAT CCC ATA GCA AGT ATC ATA G-3՛) for fragment C, OMKB616 (5՛-CTA CTG GTT TAG AAG TTG ATA C-3՛) and OMKB617 (5՛-TAC TGG AAT AGA GGA TAA CAA G-3՛) for fragment D, OMKB618 (5՛-GTT ATC CTC TAT TCC AGT AGC-3՛) and OMKB619 (5՛-CAT ACA TCC TAA CAT TAA TAA CG-3՛) for fragment E, and OMKB620 (5՛-CGC TGA CTT CCT GGC TAA AC-3՛) and OMKB541 (5՛-ATT GTT CTA CAT TAC GAG ATA CC-3՛) for fragment F.

### Immunofluorescence assay.

Synchronous parasites harboring *PfBlm*-*GFP*, *PfRad51*-*GFP*, or *PfMtDNAP* were fixed using 4% formaldehyde (Sigma). Membrane permeabilization was performed using 1:3 chilled acetone-methanol, followed by 1-h blocking using 3% bovine serum albumin (BSA). Parasites were probed with mouse anti-cytochrome *c* (Abcam) and rabbit anti-GFP (Abcam) primary antibodies for 1 h at 37°C followed by three washes with 1× PBST (PBS with Tween 20). Cells were then treated with the secondary antibody cocktail containing Alexa Fluor 488-conjugated goat anti-rabbit IgG (green), Alexa Fluor 594-conjugated rabbit anti-mouse IgG (red), and Hoechst 33342 (blue) (Invitrogen) and subsequently washed thrice with 1× PBST. Finally, parasites were mounted using antifade (Life Technologies). Fluorescence microscope Nikon Eclipse NiE AR was used for analyzing and capturing the green and red fluorescence of GFP and CytC, respectively.

### Western blotting.

Parasite proteins isolated from different subcellular fractionations were separated on SDS-polyacrylamide gels and were transferred to polyvinylidene difluoride (PVDF) membranes as previously described (48). The primary antibodies used were rabbit anti-PfRad51 (1:5,000 dilution) (25), rabbit anti-PfBlm (1:5,000) (27), rabbit anti-PfalMre11 (1:5,000) (26), rabbit anti-HsHistone 3 (1:5,000; Imperial Life Sciences), mouse anti-cytochrome *c* (1:5,000; Abcam), rabbit anti-GFP (1:5,000; Abcam), and mouse anti-GAPDH (1:5,000; Abcam). The membranes were washed with Tris-buffered saline with Tween 20 (TBS-T) and treated with horseradish peroxidase (HRP)-conjugated anti-mouse (1:10,000; Santa Cruz Biotechnology Inc.) and anti-rabbit (1:10,000; Promega) secondary antibody for 2 h at 4°C. After washes with TBS-T, membranes were developed with a chemiluminescent horseradish peroxidase (HRP) substrate (SuperSignal West Pico Plus; Thermo Scientific) and imaged by using a ChemiDoc Touch imaging system from Bio-Rad.
